# *Epimedii Herba*: An ancient Chinese herbal medicine in the prevention and treatment of rheumatoid arthritis

**DOI:** 10.3389/fchem.2022.1023779

**Published:** 2022-11-17

**Authors:** Liu-Bo Zhang, Yu Yan, Jun He, Pei-Pei Wang, Xin Chen, Tian-Yi Lan, Yu-Xuan Guo, Jin-Ping Wang, Jing Luo, Ze-Ran Yan, Yuan Xu, Qing-Wen Tao

**Affiliations:** ^1^ Department of TCM Rheumatism, Department of Pharmacy, Institute of Clinical Medical Sciences, China-Japan Friendship Hospital, Beijing, China; ^2^ China-Japan Friendship Clinical Medical College & School of Life Sciences, Beijing University of Chinese Medicine, Beijing, China; ^3^ School of Chinese Medicine, Shenyang Pharmaceutical University, Shenyang, China

**Keywords:** *Epimedii Herba*, rheumatoid arthritis, pharmacology, bioavailability, toxicity

## Abstract

Rheumatoid arthritis (RA) is a chronic, progressive inflammatory and systemic autoimmune disease resulting in severe joint destruction, lifelong suffering and considerable disability. Diverse prescriptions of traditional Chinese medicine (TCM) containing *Epimedii Herba* (EH) achieve greatly curative effects against RA. The present review aims to systemically summarize the therapeutic effect, pharmacological mechanism, bioavailability and safety assessment of EH to provide a novel insight for subsequent studies. The search terms included were “*Epimedii Herba*”, “yinyanghuo”, “arthritis, rheumatoid” and “Rheumatoid Arthritis”, and relevant literatures were collected on the database such as Google Scholar, Pubmed, Web of Science and CNKI. In this review, 15 compounds from EH for the treatment of RA were summarized from the aspects of anti-inflammatory, immunoregulatory, cartilage and bone protective, antiangiogenic and antioxidant activities. Although EH has been frequently used to treat RA in clinical practice, studies on mechanisms of these activities are still scarce. Various compounds of EH have the multifunctional traits in the treatment of RA, so EH may be a great complementary medicine option and it is necessary to pay more attention to further research and development.

## Introduction

Rheumatoid arthritis (RA) is classified as a chronic, progressive inflammatory and systemic autoimmune disease that primarily manifests as a symmetric poly-arthritis in hands and feet (Cush, 2021), leading to severe joint destruction, lifelong suffering and considerable disability (Burmester and Pope, 2017). The global prevalence of RA is estimated at approximately 1% (van der Woude and van der Helm-van Mil, 2018). In case of inadequate treatment, RA can result in permanent cartilage degradation, bone abrasions, joint impairment, impaired movement, and even irreversible disability (Chakraborty et al., 2021). Consequently, the issue of prompt and targeted RA treatment has received considerable attention. Currently, the main classes of therapeutic medications against RA are glucocorticoid, nonsteroidal anti-inflammatory drugs and disease-modifying antirheumatic drugs (DMARDs). Without doubt, those medications have greatly therapeutic effects, but they are related to harmful side effects, such as gastrointestinal bleeding, osteoporosis, stomatitis, fatigue and hepatotoxicity (Lin et al., 2020). Extensive research has shown that traditional Chinese medicine (TCM) has remarkable advantages of alleviation effectively of symptoms of RA and lowered side effects. Therefore, TCM is necessary to be seen as a complementary medicine strategy.

**FIGURE 1:**
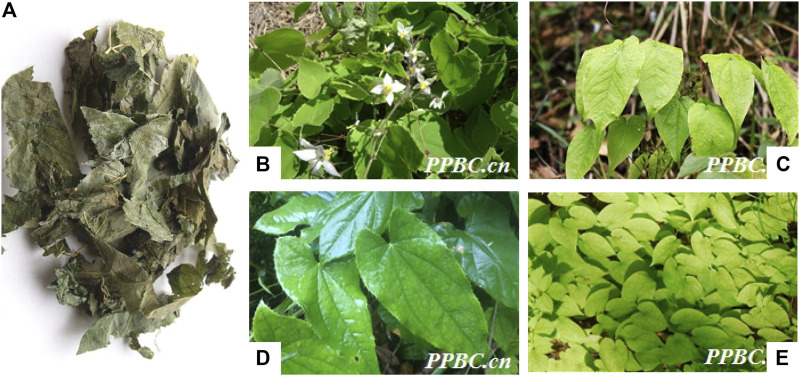
*Epimedii Herba* **(A)** (https://www.daquan.com/) is the dried leaves originated from several plants of the genus Epimedium, including *Epimedium brevicornu* Maxim **(B)**, *Epimedium sagittatum* (Sieb. EtZucc.) Maxim **(C)**, *Epimedium pubescens* Maxim **(D)** and *Epimedium koreanum* Nakaias **(E)** (Cited from plant photo bank of China at http://ppbc.iplant.cn/).

*Epimedii Herba* (EH), a classical herbal medicine, is the dried leaves originated from several plants of the genus Epimedium (Cho et al., 2017) including *Epimedium brevicornu* Maxim., *Epimedium sagittatum* (Sieb. EtZucc.) Maxim., *Epimedium pubescens* Maxim. and *Epimedium koreanum* Nakaias according to Chinese Pharmacopoeia (Figure 1). EH exerts osteoprotective effects by strengthening bones and muscle, dispelling wind chill and tonifying kidney in the Chinese medicine classics *Shen Nong Ben Cao Jing* (Yang et al., 2018a). EH alone, or combined within the TCM prescriptions has been used extensively to treat various disease including osteoporosis (Wang et al., 2016), cancer (Chen et al., 2016), chronic fatigue syndrome (Chi et al., 2017) and sexual dysfunction (Niu, 1989). Furthermore, in clinical practice, EH is one of the most frequently used herbs in a variety of traditional Chinese decoction for the treatment of RA, such as Bushen Quhan Zhiwang decoction (Wang G et al., 2021), Yishen Qubi Tongluo decoction (Luo. et al., 2019) and Bushen Jiedu Tongluo decoction (Yuan. et al., 2019). Additionally, EH is also a component of Chinese patent medicine for treating RA, including Wangbi tablet (Chen. et al., 2021), Kunxian capsule (Sun. and Chen., 2021) and Fugui Gutong capsule (Zhang. et al., 2020b). The prescriptions of TCM containing EH used by physicians for management of RA were shown in Table 1. EH had drawn increased attention and pharmacological research to explore material foundation and pharmacological mechanism for treating RA.

**TABLE 1:** The prescriptions of TCM containing EH used by physicians for management of RA.

Prescription name	Components	Effects	References
Kunxian capsule	*Epimrdii Herba* *, Cuscutae Semen, Lycii Fructus, Tripterygium hypoglaucum*	↓ESR, CRP, number of joint swelling, number of joint tenderness, morning stiffness time, VAS score	Sun. and Chen., 2021
Bushen Quhan Zhiwang decoction	*Psoraleae Fructus* 10g*, Dipsaci Radix* 15 g*, Rehmanniae Radix Praeparata* 15g*, Aconiti Lateralis Radix Praeparata* 6g*,* *Epimrdii Herba* 10 g*, Drynariae Rhizoma* 10 g*, Cinnamomi Ramulus* 9 g, *Angelicae Pubescentis Radix* 20 g*, Clematidis Radix Et Rhizoma* 15 g*, Paeoniae Radix Alba* 20 g*, Saposhnikoviae Radix* 10 g*, Atractylodis Rhizoma 15* *g, Ephedra Herba* 6 g*, Paeoniae Radix Rubra* 10 g*, Anemarrhenae Rhizoma* 10g*, Lycopodii Herba* 15 g*, Eupolyphaga Steleophaga* 10 g*, Achyranthis Bidentatae Radix* 10 g	↓ESR, CRP, RF, number of joint swelling, number of joint tenderness, morning stiffness time, HAQ	Wang Y et al. (2021)
Wangbi tablet	*Rehmanniae Radix Praeparata, Rehmanniae Radix, Anemarrhenae Rhizoma,* *Epimrdii Herba,* *Dipsaci Radix, Cibotii Rhizoma, Lycopodii Herba, Carthami Flos, Paeoniae Radix Alba, Cinnamomi Ramulus, Angelicae Pubescentis Radix, Saposhnikoviae Radix, Clematidis Radix Et Rhizoma, Aconiti Lateralis Radix Praeparata*	↓DAS28 score, TCM symptom score↑ACR20, ACR50	Chen. et al. (2021)
		
Bushen Tongluo recipe	*Epimrdii Herba* 30 g*,* *Eucommiae Cortex* 9 g*, Dipsaci Radix* 9 g*, Clematidis Radix Et Rhizoma* 27 g*, Radix Rhodomyrti* 30 g	↓TCM symptom score	Shu. and Cai., 2021
Wanbi Xinglei Yin	*Astragali Radix*30 g*, Angelicae Sinensis Radix*20 g*, Polygoni Multiflori Radix* 30 g*, Atractylodis Macrocephalae Rhizoma* 15 g*, Salviae Miltiorrhizae Radix Et Rhizoma* 20g*, Taxilli Herba* 30 g*,* *Epimrdii Herba* 15 g*,* *Acanthopanacis Cortex* 15g *, Manis pentadactyla* 10g*, Zaocys* 12g*, Speranskia tuberculate* 30g*, Glycyrrhizae Radix Et Rhizoma* 9g	↓VAS score, HAQ, number of joint swelling, number of joint tenderness, morning stiffness time	Zhang. et al. (2020a)
Fugui Gutong capsules	*Aconiti Lateralis Radix Praeparata, Aconiti Radix Cocta, Cinnanmomi Cortex, Codonopsis Radix, Angelicae Sinensis Radix, Paeoniae Radix Alba,* *Epimrdii Herba,* *Olibanum*	↓ESR, CRP, number of joint swelling, number of joint tenderness, morning stiffness time, IL-1, IL-6, TNF-α	Zhang. et al. (2020b)
Yishen Qubi tongluo decoction	*Epimrdii Herba* 10g*,* *Psoraleae Fructus* 10 g*, Dipsaci Radix* 12 g*, Rehmanniae Radix Praeparata* 10g*, Notopterygii Rhizoma Et Radix* 1 5g*, Angelicae Pubescentis Radix* 15 g*, Cinnamomi Ramulus* 10g*, Sinomenii Caulis* 15 g*, Olibanum* 5 g*, Myrrha* 5 g*, Angelicae Dahuricae Radix* 10 g*, Clematidis Radix Et Rhizoma* 10 g*, Astragali Radix* 15 g*, Angelicae Sinensis Radix* 15 g	↓TCM symptom score	Luo. et al. (2019)
Bushen Jiedu Tongluo decoction	*Scorpio* 3 g*, Glycyrrhizae Radix Et Rhizoma* 6g*, Notopterygii Rhizoma Et Radix* 9g*, Moutan Cortex* 9 g*, Paeoniae Radix Alba* 9 g*, Zaocys* 9 g*, Curculiginis Rhizoma* 15 g*,* *Epimrdii Herba* 15g*,* *Dipsaci Radix* 15 g*, Sinomenii Caulis* 15 g*, Rehmanniae Radix* 15 g*, Curcumae Longae Rhizoma* 15 g*, Lonicerae Japonicae Caulis* 30 g*, Sarcandrae Herba* 30g	↓ESR, CRP, RF, number of joint swelling, number of joint tenderness, VAS score, HAQ, IL-1, DAS28	Yuan. et al. (2019)
Tonifying Liver and Kidney decoction	*Lycopodii Herba* 20 g*, Dipsaci Radix* 20 g*,* *Epimrdii Herba* 15 g*,* *Drynariae Rhizoma* 15 g*, Psoraleae Fructus* 15 g*, Spatholobi Caulis* 15 g*, Paeoniae Radix Alba* 1 2g*, Cinnamomi Ramulus* 12 g*, Angelicae Pubescentis Radix*12g*, Achyranthis Bidentatae Radix* 12 g	↓number of joint swelling, number of joint tenderness, morning stiffness time, hs-CRP, ESR, RF, ADL score, QOL score	Liang. et al. (2019)
Kidney-tonifying arthralgia-eliminating decoction	*Aconiti lateralis Radix Praeparaia* 6 g*, Dipsaci Radix* 10 g*, Eucommiae Cortex* 10g*, Achyranthis Bidentatae Radix* 10 g*, Manis pentadactyla* 9 g*,* *Epimrdii Herba* 8g*,* *Hirudo* 6g*, Speranskia tuberculate* 20 g*, Rhizoma Seu Herba Aristolochiae Mollissimae* 10g*, Pyritum* 6g	↓number of joint swelling, number of joint tenderness, morning stiffness time, CRP, ESR, RF, FG, VEGF	Liu and Li (2018)
Xianzhi Fengsui decoction	*Corni Fructus*30 g*,* *Epimrdii Herba* 15 g*,* *Psoraleae Fructus* 15 g*, Phellodendri Chinrnsis Cortex* 10 g*, Amomi Fructus* 6 g*, Glycyrrhizae Radix Et Rhizoma* 6 g	↓number of joint tenderness, morning stiffness time, ESR, RF, BMD, OPG	Zheng. et al. (2017)

TCM, traditional Chinese medicine; VAS, visual analogue scale; HAQ, health assessment questionnaire; ESR, erythrocyte sedimentation rate; CRP, C-reactive protein; RF, rheumatoid factor; DAS28, Disease Activity Score for 28 joints; ACR20, American College of Rheumatology 20%; ADL, daily living ability; QOL, life therapy scale; FG, fibrinogen; VEGF, vascular endothelial growth factor; BMD, bone mineral density; OPG, osteoprotegerin.

**FIGURE 2:**
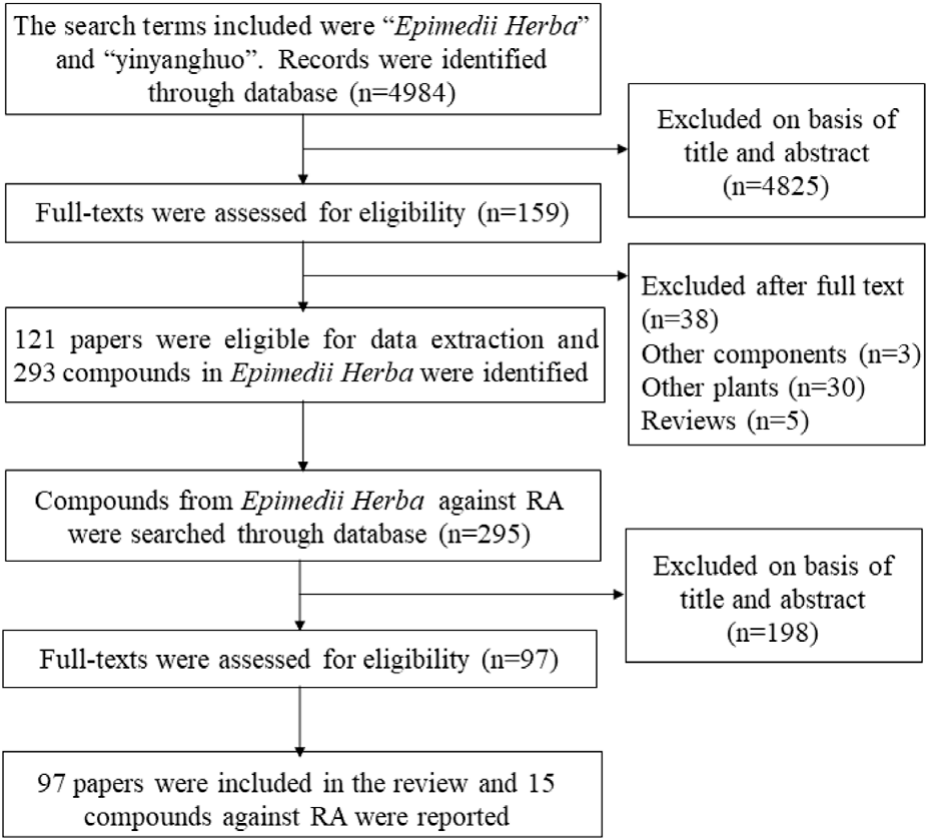
Articles were identified and screened for eligibility.

Sze et al. have reviewed anti-oxidative properties of EH (Sze et al., 2010). However, the study did not review anti-inflammatory, immunoregulatory, osteoprotective and antiangiogenic activities. Consequently, the information of EH was searched to review the current research status. In detail, the literatures on compounds from EH were obtained using Web of Science, Google Scholar, Pubmed, and CNKI. Subsequently, the literatures on compounds involved in RA treatment were further filtered (Figure 2). Based on various pathological mechanism of RA including inflammation of the synovial membrane, oxidation, angiogenesis and bone destruction, this article systematically summarizes anti-RA activities of EH and further explores material basis and mechanism of these activities, so as to provide novel insight in the treatment of RA.

## Effects of EH on RA

### EH and its total flavonoids

*In vivo* studies were performed on adjuvant-induced arthritis (AIA) mice to investigate the anti-RA effects after an oral administration of EH and the results showed that compared with *Tripterygii Radix*, paw thickness was lower after oral administration of EH combined with *Tripterygii Radix* (Du. et al., 2019). It was reported that total flavonoids of EH can inhibit differentiation and bone resorption of osteoclasts (Zhang et al., 2012). Furthermore, total flavonoids of EH were found to promote osteogenic differentiation *via* the bone morphogenetic protein and Wnt/β-catenin signaling pathways (Zhang et al., 2010).

### Components of EH

**FIGURE 3:**
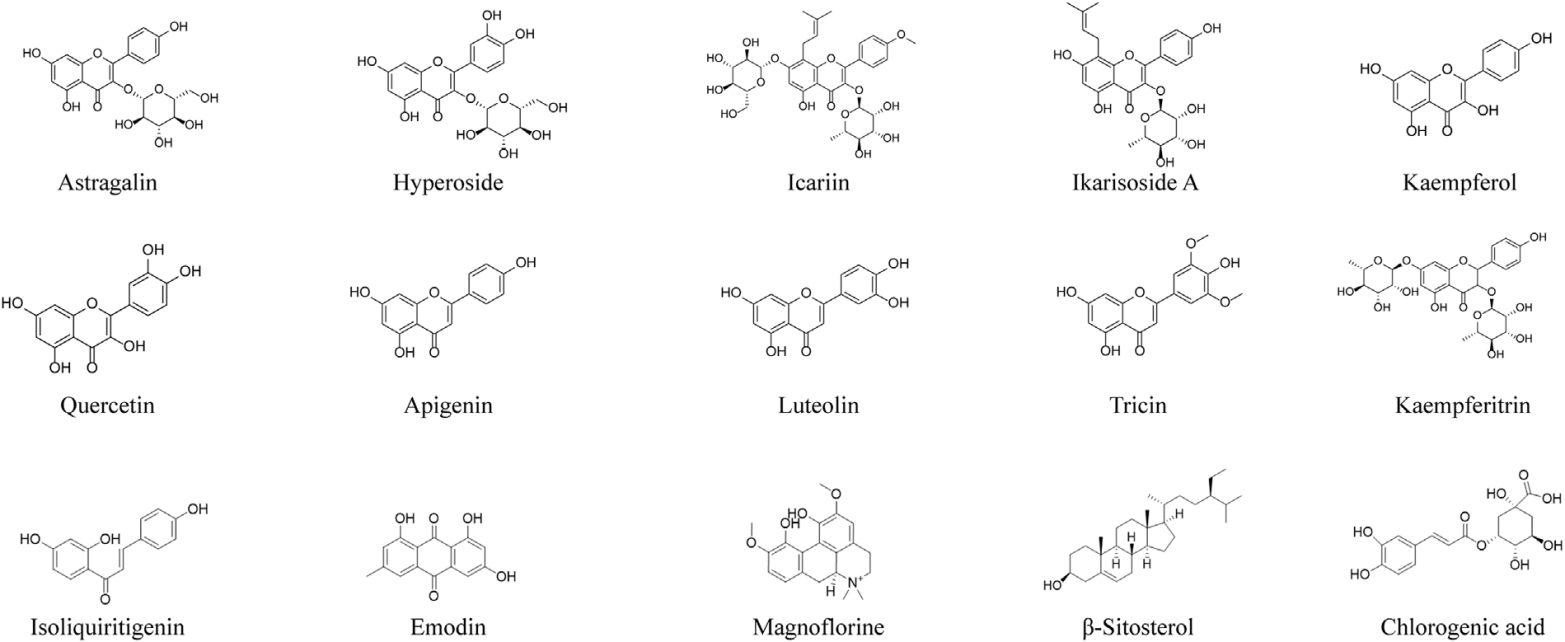
Structure of compounds from EH against RA.

293 compounds from EH were searched in different database (Ma et al., 2011; Jin et al., 2014; Li F. et al., 2017; Pang et al., 2018; Ren et al., 2018; Su et al., 2018; Zhang et al., 2020; Hu. et al., 2021). Then, the studies of the compounds against RA were further searched in the database and 15 compounds for the treatment of RA have been reported, including icariin, quercetin, kaempferol, apigenin, luteolin, kaempferitrin, astragalin, hyperoside, ikarisoside A, tricin, isoliquiritigenin, emodin, *β*-sitosterol, magnoflorine and chlorogenic acid (Figure 3). Icariin is the most abundant constituent in EH and one of chemical markers for quality control of EH. Other compounds extracted from EH may have positive effects for the treatment of RA. The overview of the compounds for the treatment of RA was shown in Table 2.

**TABLE 2:** Compounds from EH for the treatment of RA.

NO.	Compounds	Subgroup	Formula	Molecular weight	Sources	Content (mg/g)	References
1	Magnoflorine	Alkaloids	C_20_H_24_NO_4_	341.1695	a	1.85 ± 1.16	Ma. et al. (2019); Cheng. et al. (2022)
2	Emodin	Anthraquinone	C_15_H_10_O_5_	270.9787	a,b	—	Cui. et al. (2010); Ma. et al. (2019)
3	Astragalin	Flavonoids	C_21_H_20_O_11_	448.0956	a, d	—	Cheng. et al. (2006)
4	Hyperoside	Flavonoids	C_21_H_20_O_12_	464.0907	a, b, c, d	0.55–2.25	Zheng. and Kong., 2002; Sun. et al. (2018); Ma. et al. (2019); Han., 2022
5	Icariin	Flavonoids	C_33_H_40_O_15_	676.1256	a, b, c, d	7.86 ± 3.74	Wang et al. (2007); Kim and Shim, (2019); Ma. et al. (2019); Yang et al. (2020); Cheng. et al. (2022)
6	Ikarisoside A	Flavonoids	C_26_H_28_O_10_	500.1623	a, c, d	—	Li et al. (1995a); Zhang. et al. (2013); Ma. et al. (2019)
7	Kaempferol	Flavonoids	C_15_H_10_O_6_	286	b, c	—	Wang et al. (2007); Zhang. et al. (2013)
8	Quercetin	Flavonoids	C_15_H_10_O_7_	302.7	b, d	—	Li. et al. (1995b); Wang et al. (2007); Hu. et al. (2021)
9	Apigenin	Flavonoids	C_15_H_10_O_5_	-	b	—	Wang et al. (2007)
10	Luteolin	Flavonoids	C_15_H_10_O_6_	286.0408	a, b, d	—	Wang et al. (2007); Ma. et al. (2019)
11	Tricin	Flavonoids	C_17_H_14_O_7_	330	b, c	—	Cui. et al. (2010); Zhang. et al. (2013)
12	Kaempferitrin	Flavonoids	C_27_H_30_O_14_	578.1590	a	—	Ma. et al. (2019)
13	Isoliquiritigenin	Chalcone	C_15_H_12_O_4_	-	d	—	Li. et al. (1994)
14	β-Sitosterol	Phytosterol	C_29_H_50_O	-	b	—	Cui. et al. (2010)
15	Chlorogenic acid	polyphenol	C_16_H_18_O_9_	354.0882	a	0.47 ± 0.38	Ma. et al. (2019); Cheng. et al. (2022)

a: *Epimedium brevicornu* Maxim. b*: Epimedium sagittatum* (Sieb. EtZucc.) Maxim. c*: Epimedium pubescens* Maxim d*: Epimedium koreanum* Nakaias.

## Anti-inflammatory activities

RA is an inflammatory disease and pro-inflammatory cytokines and chemokines are overproduced in synovial fluid and serum of RA patients (Shrivastava and Pandey, 2013; Jiang et al., 2021). It was reported that astragalin, hyperoside, kaempferol, icariin, apigenin and kaempferitrin treatment decreased arthritic score and incidence of arthritis in animal models (Chi et al., 2014; Li et al., 2016; Jin et al., 2017; Lee et al., 2018; Jia et al., 2019a; Wang and Zhao, 2019; Lei. et al., 2020; Jin. et al., 2021). Furthermore, arthritis symptoms including paw volume and paw thickness were reduced significantly with quercetin, astragalin, hyperoside, icariin, luteolin, kaempferol and kaempferitrin treatment in animal models of arthritis (Haleagrahara et al., 2017; Pan et al., 2018; Jia et al., 2019b; Wang and Zhao, 2019; Lei. et al., 2020; Ahmed and Abd Elkarim, 2021; Jin. et al., 2021; Liu Y et al., 2021). The anti-arthritis effect of compounds from EH in the animal models was shown in Table 3. Anti-arthritis mechanism of compounds extracted from EH may be related to inhibiting inflammatory mediators, such as cytokines, chemokines, Prostaglandin E2 (PGE2), cyclooxygenase (COX)-2, nitric oxide (NO) and inducible nitric oxide synthase (iNOS).

**TABLE 3:** The anti-arthritis effect of compounds from EH for the treatment of RA in the animal models.

Compounds	Model	Dose (mg/kg)	Action	References
Apigenin	CAIA mice	16	↓paw volume, paw thickness, arthritic score	Jin et al. (2017)
	CIA mice	20	↓arthritis incidence, joint swelling, clinical scores	Li et al. (2016)
Astragalin	CIA mice	5	↓arthritis index, swollen joints count, paw thickness	Jia et al. (2019a)
Chlorogenic acid	CIA mice	60	↓arthritis index, paw thickness	Fu et al. (2019)
Emodin	AIA mice	30	↓paw edema, arthritis scores	Zhu et al. (2019)
	CIA mice	20	↓paw thickness, arthritis index, incidence, clinical scores	Hwang et al. (2013); Zhu et al. (2013)
Hyperoside	CIA mice	50	↓paw thickness, arthritis index	Jin. et al. (2021)
Icariin	AIA rabbits	60	↓mankin score	Wei et al. (2016)
	CIA mice	40	↓arthritic score, swollen joints	Lei. et al. (2020)
	CIA mice	25	↓arthritis incidence	Chi et al. (2014)
Kaempferitrin	CIA mice	20	↓paw thickness, arthritis scores	Wang and Zhao, (2019)
Kaempferol	CIA mice	200	↓disease severity, joint swelling	Pan et al. (2018)
	CIA mice	2	↓arthritis severity, arthritis incidence	Lee et al. (2018)
	AIA mice	25	↓paw volume	Ahmed and Abd Elkarim, (2021)
Luteolin	CIA mice	100	↓paw volume	Liu Y. et al. (2021)
Magnoflorine	AIA mice	10	↓arthritis scores, paw swelling. ↑ body weight	Shen et al. (2022)
Quercetin	CIA mice	30	↓paw oedema	Haleagrahara et al. (2017)
*β*-Sitosterol	CIA mice	100	↓arthritis index, ankle swelling, paw thickness	Liu et al. (2019); Qian et al. (2021)

CIA, Collagen-induced arthritis; CAIA , Collagen antibody-induced arthritis., AIA, antigen-induced arthritis.

### Effects on inflammatory cytokines

Proinflammatory cytokines including tumor necrosis factor-α (TNF-α), interleukin-6 (IL-6), IL-1β are responsible for the induction and maintenance of inflammatory milieu in the synovial tissue and articular destruction (Mateen et al., 2016b). Especially TNF-α, a central cytokine in RA pathophysiology (Feldmann et al., 1996), stimulates activation of leukocyte, endothelial, stromal-cell and chondrocyte, as well as expression of angiogenesis, nociception, chemokine (McInnes et al., 2016). Additionally, TNF-α directly increases osteocyte receptor activator of NF-κB ligand (RANKL) expression and induces formation of osteoclasts (Marahleh et al., 2019). Blocking bioactivity of TNF-α reduces production of IL-1, IL-1β, IL-6 and IL-8 (Brennan and McInnes, 2008). Consequently, TNF blockers are one of the most valuable agents to prevent bone erosion and loss in RA.

*In vivo* and *in vitro* studies showed that icariin, quercetin and kaempferitrin reduced the expression of IL-1β, IL-6 and TNF-α *via* PI3K/AKT/Mtor (Wang and Zhao, 2019; Xiang. et al., 2020), miR-223-3p/NLRP3 (Lei. et al., 2020; Wu et al., 2020), and NF-κB signaling pathway (Jia. et al., 2019a; Wang Z et al., 2019). Moreover, luteolin, tricin, kaempferol, isoliquiritigenin, emodin, astragalin and hyperoside inhibited proinflammatory cytokines including IL-1β, IL-6, TNF-α, IL-8, IL-17 and IL-1 (Kim et al., 2008; Hwang et al., 2013; Jin et al., 2016; Jia et al., 2019a; Zhu et al., 2019; Ji et al., 2021; Lin et al., 2021; Ling et al., 2021; Zhou et al., 2021). Apigenin suppressed bone marrow-derived dendritic cells (DCs) to produce cytokines including TNF-α, IL-12 p70 and IL-10, while increased the secretion of IL-1β (Li et al., 2016). Haque et al. reported that magnoflorine enhanced pro-inflammatory responses through producing of TNF-α and IL-1β (Haque et al., 2018). However, Shen et al. showed that magnoflorine attenuated inflammatory responses by reducing inflammatory cytokines, such as IL-6 and IL-8 (Shen et al., 2022). Further research is needed to confirm this opposite conclusion.

### Effects on the production of chemokines

Previous research has confirmed that various chemokines are up-regulated in serum, synovial fluid and synovial tissue of patients with RA, compared with healthy controls (Miyabe et al., 2020). Furthermore, chemokines including CXCL13 and CXCL10 can be considered as biomarkers of RA disease activity (Meeuwisse et al., 2011; Pandya et al., 2017). In RA, chemokines boost neutrophils, T cells and the B cells recruitment into the joint (Lee et al., 2017; Miyabe et al., 2017; Armas-Gonzalez et al., 2018). Targeting chemokines might be a promising direction for RA therapies.

It was reported that quercetin suppressed expression of monocyte chemoattractant protein (MCP)-1 in the collagen-induced arthritis (CIA) and AIA mice (Gardi et al., 2015; Haleagrahara et al., 2017). Apigenin inhibited arthritis development through reducing the migration of DCs, which may be related to down-regulating the expression of chemokine receptor 4 (CXCR4) (Li et al., 2016).

### Effects on the production of PGE2 and COX

PGE2, a main mediator of inflammation in RA, contributes to the pathogenesis of RA. It can be produced by diverse immune cells upon the activation of COX enzymes and PGE synthases (Park et al., 2006). PGE2 increased IL-17 production and promoted the migration and antigen-presenting function of DCs (Okano et al., 2006; Sheibanie et al., 2007). Furthermore, PGE2 suppressed regulatory T (Treg) cells differentiation *via* the EP2-cAMP/PKA signaling pathway (Li H. et al., 2017).

Quercetin and kaempferol remarkably suppressed production of PGE2 in human rheumatoid arthritis fibroblast-like synoviocytes (RAFLS), and the mechanism might be related to the inhibition of COX1 and COX2 expression (Lee and Kim, 2010; Sung et al., 2012; Yoon et al., 2013). PGE2 and COX2 were suppressed by quercetin, emodin and icariin in RA mice (Zhu et al., 2013; Yang et al., 2018b; Guazelli et al., 2018; Liu T et al., 2021). Apigenin, isoliquiritigenin and luteolin treatment had an inhibitory effect on the expression of COX2 in lipopolysaccharide (LPS) induced RAW 264.7 macrophage cells (Kim et al., 2008; Lee and Kim, 2010).

### Effects on the production of NO and iNOS

Overexpression of iNOS increases the levels of NO, which is an important mediator of synovial inflammation in RA (Li and Wan, 2013; Minhas et al., 2020).

Serum level of NO was effectively decreased in CIA mice with quercetin treatment (Choi et al., 2009). Additionally, apigenin, luteolin, quercetin and kaempferol had an inhibitory effect on NO production stimulated by LPS in RAW 264.7 macrophage cells (Lee and Kim, 2010). *In vitro* studies showed that magnoflorine, isoliquiritigenin, quercetin, kaempferol, luteolin and apigenin suppressed the level of iNOS (Sun et al., 2020; Lin et al., 2021) and the mechanism may be associated with PI3K/Akt/NF-κB signaling axis and the Keap1-Nrf2/HO-1 signaling pathway (Shen et al., 2022). Furthermore, iNOS was inhibited markedly by quercetin in CIA mice (Yang et al., 2018b) and suppressed by hyperoside in fibroblast-like synoviocytes (FLS) (Fu. et al., 2020).

## Immunoregulatory activity

RA is a chronic autoimmune disease characterized as infiltration of the synovial membrane in multiple joints with immune cell such as T cells, B cells, macrophages and DCs (Aletaha and Smolen, 2018). Compounds from EH have immunoregulatory effects *via* regulating T cells, macrophages, neutrophils, DCs and B cells.

Under antigenic stimulation and cytokine signaling, naive CD4^+^ T cells activate and differentiate into various Th cell subsets, including Th1, Th2, Th17, follicular Th, and Treg cells. Th1/Th2 and Th17/Treg cells become disproportional in the pathogenesis of RA (Noack and Miossec, 2014; Wang Y et al., 2019). Th1 cells secrete pro-inflammatory factors including interferon-γ (IFN-γ) and TNF-α and Th2 cells secrete anti-inflammatory factors, such as IL-4, IL-10 and IL-13 (Srivastava et al., 2018). Th17 cells accelerate secretion of IL-17 that stimulates production of proinflammatory cytokines including TNF-α, IL-1β, IL-6, matrix metalloproteinases (MMPs) and chemokines (Jin et al., 2018). Treg cells exert anti-inflammatory effects *via* secreting IL-10 and transforming growth factor-β (TGF-β) (Jiang et al., 2021). Sun et al*.* found that luteolin and apigenin treatment significantly reduced levels of IFN-γ and IL-2 in concanavalin A -induced splenic T-lymphocyte (Sun et al., 2020). Recent studies have shown that apigenin and chlorogenic acid regulated Th1/Th2 cells balance by inhibiting cytokines of Th1 cells and elevating Th2 cytokines (You. et al., 2009; Chauhan et al., 2012; Zhang. et al., 2015). Quercetin and kaempferol modulated the balance between Th17 and Treg immune response by downregulating Th17 cells and upregulating Treg cells (Lin et al., 2015; Yang et al., 2018b; Lee et al., 2018). Icariin treatment reduced number of Th17 cells in mouse spleens and synovial and suppressed Th17 cell differentiation *in vitro* experiments *via* downregulating STAT3 activation (Chi et al., 2014).

Macrophages are commonly divided into two distinct phenotypes, called M1-like macrophages and M2-like macrophages. M1 macrophages are considered as a pro-inflammatory phenotype *via* expressing pro-inflammatory cytokines and chemokines, while M2 macrophages release anti-inflammatory cytokines including IL-10 and TGF-β (Ross et al., 2021). The number of active M1 macrophages was increased in RA patients while the number of M2 type cells was decreased or inactive (Kennedy et al., 2011). Therefore, blocking M1 macrophage-derived cytokines and modulating the balance between M1 macrophages and M2 macrophages may contribute to the improvement of RA. Icariin and *β*-sitosterol suppressed M1 macrophage activation and increased M2 macrophage activation (Liu et al., 2019) and the mechanism may be related to inhibit mTOR/S6K1 and NF-κB signaling (Li et al., 2011). *In vivo* and *in vitro* studies showed that quercetin inhibited macrophage-derived NO, TNF-α, IL-1β and MCP-1 (Mamani-Matsuda et al., 2006).

In the absence of inflammation, healthy neutrophils circulate in the blood within several hours and undergo apoptosis (McCracken and Allen, 2014). In RA, however, neutrophils inappropriately activated by autoantibodies and inflammatory mediators are characterized by a delayed apoptotic process and migration into the joint (Cecchi et al., 2018). In addition, neutrophils produce reactive oxygen species (ROS) and release diverse cytokines and chemokines, contributing to inflammation and tissue damage (Fresneda Alarcon et al., 2021). The RA synovial microenvironment results in the formation of neutrophil extracellular traps (NETs) that are a source of citrullinated autoantigens and activate FLS, accelerating disease progression and joint damage in RA (Carmona-Rivera et al., 2017). Thus, NETs formation and neutrophil-produced cytokines, chemokines are considered as novel treatment targets (O'Neil and Kaplan, 2019). Quercetin suppressed neutrophil infiltration and NETs formation and increased the apoptosis of activated neutrophils in AIA mice (Yuan et al., 2020). Emodin reduced neutrophil infiltration in AIA mice and increased apoptosis and inhibited autophagy and NETs *in vitro* (Zhu et al., 2019).

DCs are antigen-presenting cells that link innate and adaptive immune responses. In RA, high concentrations of DCs are recruited in joint synovial fluid and tissues and DCs within the RA synovium are generally mature (Wehr et al., 2019). A recent study showed that synovial microenvironment in RA was responsible for DCs maturation and metabolic reprogramming *via* up-regulating STAT3 activation (Canavan et al., 2020). In addition, intracellular Zn^2+^ homeostasis and low oxygen state also impacted the maturation of DCs (Qiao et al., 2022). Inhibition of DCs maturation is an important treatment of DC-targeting in RA. *In vivo* and *in vitro* studies showed that apigenin efficiently inhibited DCs maturation and reduced cytokine secretion (Li et al., 2016).

The functions of B cells are closely associated with the pathogenesis of RA, such as antigen presentation, cytokine secretion and autoantibody production (Wu et al., 2021). B cells present autoantigens to T cells and secrete various cytokines including TNF-α, IFN-γ, IL-6, IL-1β, IL-17 and IL-10 (Yanaba et al., 2008). B-cell activating factor (BAFF), a member of the TNF superfamily, promoted the differentiation, proliferation, and activation of B cells *via* NF-κB signaling pathway (Zhang et al., 2021). Compared with healthy individuals, patients with RA had higher levels of BAFF in the peripheral blood and synovial fluid (Moura et al., 2011). Chlorogenic acid inhibited BAFF Expression in CIA mice and MH7A cells through the NF-κB pathway (Fu et al., 2019).

## Osteoprotective activities

Bone and cartilage destruction can be evaluated by morphology, histology, X-ray, and computed tomography scan. Astragalin significantly reduced joint space widening and synovial vascularity by ultrasonography and color doppler and markedly diminished bone destruction of knee and ankle joints by the 3D reconstruction of a micro-CT analysis (Jia et al., 2019b). Icariin inhibited trabecular bone loss and increased bone mineral density in AIA rabbits by micro-CT analysis (Wei et al., 2016). Quercetin treatment attenuated level of ^8^F-FDG in the ankle and knee joints of CIA mice by 18F-FDG micro-PET imaging, suggesting it could reduce inflammation in joints (Shen et al., 2021). Furthermore, bone erosion and degradation were not serious and the narrowing of joint space was slight with quercetin treatment compared with the arthritis group in the X-ray examination (Haleagrahara et al., 2017). The compounds from EH play osteoprotective roles by decreasing formation and differentiation of osteoclasts, regulating of RANKL/osteoprotegerin (OPG) ratio and downregulating MMPs.

### Effects on formation and differentiation of osteoclasts

Formation and differentiation of osteoclasts are the essential elements of bone degradation. Osteoclast differentiation is regulated by the molecular triad RANKL, receptor activator of nuclear factor-κB (RANK) and OPG (Aureal et al., 2020). RANKL-RANK signaling activates osteoclast differentiation and suppresses osteoclast apoptosis (Kitaura et al., 2020). OPG, a RANKL decoy receptor, can prevent RANKL-RANK binding (Boyle et al., 2003). The ratio of RANKL to OPG can be regarded as a marker of progression of osteoclast destruction (van Tuyl et al., 2010).

Ikarisoside A and isoliquiritigenin suppressed osteoclastogenesis in RANKL-stimulated RAW 264.7 cells and bone marrow-derived macrophages *via* MAPK and NF-κB pathways (Choi et al., 2010; Zhu et al., 2012). Furthermore, *in vivo* and *in vitro* studies showed that isoliquiritigenin had an anti-osteoclastogenic activity by suppressing NF-κB-dependent autophagy (Liu et al., 2016).

Emodin inhibited the osteoclast differentiation in bone marrow macrophages (Hwang et al., 2013). Quercetin attenuated IL-17-induced RANKL expression in RAFLS (Kim et al., 2019). Several *in vivo* models suggested that icariin decreased osteoclasts formation, mechanism of which might be relate to the regulation of RANKL/OPG ratio (Liu. et al., 2013; Wei et al., 2016). Apigenin and luteolin treatment regulated the RANKL/OPG ratio in CIA mice by inhibiting RANKL expression and elevating OPG expression (Liu and Li, 2018; Li et al., 2019).

### Effects on cartilage protection

MMPs, belonging to the proteolytic enzymes, are intimately involved in degradation of extracellular matrix in cartilage (Itoh, 2017). Tissue inhibitor of metalloproteinases (TIMPs), a natural inhibitor, specifically inhibit MMPs (Alamgeer et al., 2020). In RA, cytokines promote chondrocytes to secret more cytokines and MMPs that degrade the cartilage and suppress generation of TIMPs (Fang et al., 2020). Furthermore, synovial tissue of RA patients produces diverse MMPs, such as MMP-1, -2, -3, -8, -9, -10, -12, -13 (Itoh, 2017). Previous studies have reported that the serum concentrations of MMP-3 can be considered as predictive marker of inflammation and joint destruction (Yamanaka et al., 2000; Shinozaki et al., 2007).

*In vivo* and *in vitro* studies showed that astragalin and quercetin inhibited the expression of MMPs *via* NF-κB pathway (Jia. et al., 2019b; Wang Z et al., 2019; Wang and Zhao, 2019) and Akt/mTOR pathways (Wang and Zhao, 2019). *In vitro* studies reported that MMPs were reduced by icariin (Chi et al., 2014), hyperoside (Fu. et al., 2020), kaempferitrin (Jia et al., 2019a), ikarisoside A, kaempferol, apigenin and luteolin treatment (Zhou et al., 2021) *via* NF-κB and MAPK signaling pathway (Choi et al., 2010; Choi and Lee, 2010; Yoon et al., 2013; Jin et al., 2016) and PI3K/Akt pathways (Hou et al., 2009). MMPs were suppressed with emodin treatment in CIA mice through NF-κB pathway (Hwang et al., 2013).

## Effects on FLS proliferation, migration and apoptosis

In RA, FLS results in hyperplasia of the synovial lining, pannus formation, joint destruction through producing cytokines, chemokines, and matrix-degrading molecules and migrating and invading joint cartilage (Bustamante et al., 2017). RAFLS are resistant to apoptosis resulting from up-regulation of anti-apoptotic mediators including Bcl-2, Mcl-2, and FLICE-inhibitory protein (FLIP) and down-regulation of pro-apoptotic proteins including tumor necrosis factor-related apoptosis-inducing ligand and p53 up-regulated modulator of apoptosis (Zhang Q. et al., 2019). Furthermore, FLS produce several enzymes connected with invasive activities of FLS, such as collagenases, aggrecanases, cathepsins, and RANKL (Tu et al., 2018). Expansion of FLS increases oxygen consumption in synovium and forms a hypoxic environment, contributing to synovium angiogenic processes and pannus formation. FLS also stimulate overproduction of MMPs including MMP1, MMP3 and MMP13, leading to degradation of the collagen-rich structures of extracellular matrix (Nygaard and Firestein, 2020). Consequently, FLS can be regarded as hopeful therapeutic target for the treatment of RA (Aletaha and Smolen, 2018).

Icariin, kaempferol, kaempferitrin, chlorogenic acid and apigenin enhanced apoptosis and restrained proliferation of RAFLS *via* regulating miR-223-3p/NLRP3 signaling pathway (Wu et al., 2020), cell cycle and mitochondrial pathway (Pu et al., 2021), MAPK pathway (Yoon et al., 2013), NF-κB pathways (Yoon et al., 2013; Lou et al., 2015; Wang and Zhao, 2019), JAK/STAT pathways (Lou et al., 2015) and PI3K/Akt/mTOR signaling pathway (Sun et al., 2012; Wang and Zhao, 2019). Quercetin elevated apoptosis and decreased the migration and invasion of FLS through mitochondrial pathway and p53 phosphorylation (Xiao et al., 2013), PI3K/Akt pathway (Pan et al., 2016) and miR-146a/GATA6 axis (Zhao et al., 2020). Kaempferol, hyperoside and luteolin had suppressive effects on either migration or proliferation of FLS by TNF signaling pathway (Ling et al., 2021), fibroblast growth factor receptor 3–ribosomal S6 kinase 2 signaling pathway (Lee et al., 2018), MAPK pathway (Hou et al., 2009; Fu. et al., 2020), NF-κB signaling pathway (Lou et al., 2015; Jin et al., 2016), PI3K/Akt pathways (Hou et al., 2009) and JAK/STAT signaling pathways (Lou et al., 2015). Magnoflorine inhibited migration, invasion, proliferation and induced apoptosis and cell cycle arrest of RAFLS *via* inhibiting the PI3K/Akt/NF-κB axis signaling pathway and activating the Keap1-Nrf2/HO-1 signaling pathway (Shen et al., 2022).

## Antiangiogenic activities

Angiogenesis, the formation of new capillaries, is related to leukocyte ingress into the synovium, synovial hyperplasia and pannus formation (Wang Z et al., 2021). Angiogenesis can be induced by angiogenic mediators including various growth factors, cytokines, chemokines, cell adhesion molecules, etc (Bodolay et al., 2002). Vascular endothelial growth factor (VEGF), one of the most vital growth factors, has a mitogenic and an anti-apoptotic effect on endothelial cells and increases the vascular permeability and cell migration (Melincovici et al., 2018). VEGF is activated by hypoxia and hypoxia-inducible factors 1 (HIF-1) and HIF-2 and pro-inflammatory cytokines including TNF-α and IL-1 (Szekanecz and Koch, 2009).

Histological evaluation demonstrated that quercetin reduced pannus formation in CIA mice (Kawaguchi et al., 2019), the mechanism of which might be associated with inhibition of VEGFA, HIF-1α and capillaries density in synovial tissue of CIA mice (Chu. et al., 2021). The expression of VEGF, VEGFR1 and VEGFR2 in synovial tissues of CIA mice and vascular cell adhesion molecule (VCAM) in human umbilical vein endothelial cells (HUVECs) were significantly inhibited by apigenin (Li et al., 2019; Zhou et al., 2021). A recent study showed that luteolin suppressed the expression of VEGF and HIF-1α in CIA mice and HUVECs (Liu T et al., 2021; Zhou et al., 2021). *In vivo* and *in vitro* studies showed that *β*-sitosterol significantly inhibited the expression and phosphorylation of VEGFR2 (Qian et al., 2021).

## Antioxidant activities

### Effects on ROS production and mitochondria dysfunction

Generally, it has been reported that oxidative stress is an associated factors in the pathogenesis of RA (Phull et al., 2018). Oxidative stress arises when enhancement of ROS exceeds the normal physiological values (Smallwood et al., 2018). The excessive production of ROS contributes to inflammation, matrix degradation and chondrocytes apoptosis *via* MAPKs and NF-κB signaling pathway (Phull et al., 2018). Mitochondria are generally regarded as the source of ROS in animal cells and mitochondrial dysfunction is responsible for imbalance of antioxidant systems (Munro and Treberg, 2017).

*In vivo* studies showed that ROS production was inhibited with quercetin and kaempferol treatment (Santos et al., 2014; Saccol et al., 2020). Apigenin induced intracellular ROS production in MH7A cells, which was associated with activation of ERK1/2 and apoptosis (Shin et al., 2009). Quercetin improved impaired mitochondrial biogenesis and mitochondrial function in CIA mice *via* regulating the SIRT1/PGC-1α/NRF1/TFAM pathway (Shen et al., 2021). *In vitro* studies showed that icariin and quercetin treatment induced apoptosis through mitochondrial pathway (Xiao et al., 2013; Pu et al., 2021).

### Effects of lipid peroxidation and myeloperoxidase activity

Previous studies have reported that RA patients have increased lipid peroxidation in the synovial fluid and blood serum (Mateen et al., 2016a). Malondialdehyde and thiobarbituric acid-reactive substance (TBARS) are widely used to measure lipid peroxidation (Ghani et al., 2017; Tsikas, 2017). 15-lipoxygenase, a lipid-peroxidizing enzyme, was largely expressed by macrophages, neutrophils and mast cells in RA synovium (Gheorghe et al., 2009).

Luteolin suppressed 15-lipoxygenase in RAW 264.7 macrophage cells (Lee and Kim, 2010). The augmentation in TBARS levels and 15-lipoxygenase were reversed with quercetin treatment (Lee and Kim, 2010; Saccol et al., 2020). Furthermore, other study reported that 12/15-lipoxygenase in lung and liver were inhibited with quercetin treatment in AIA mice (Gardi et al., 2015). Quercetin and kaempferol treatment reduced the myeloperoxidase activity in neutrophils (Santos et al., 2014).

## Clinical trials

Although various compounds extracted from EH exert anti-RA effect, few compounds have been used in clinical. In a randomized controlled trial, compared to azathioprine plus placebo or lower doses of quercetin (500, 1000 mg/day), azathioprine plus quercetin (1500 mg/day) in patients with RA obviously downregulated IL-6, intercellular adhesion molecule-1, complement proteins and upregulated IL-10 (Al-Rekabi et al., 2015). Quercetin supplement (500 mg/day) for 8 weeks exerted the beneficial effects on pain, stiffness, disease activity, inflammatory factors and well-being in women with RA in a double-blind, randomized controlled trial (Javadi et al., 2017). However, another study reported that quercetin (500 mg/day) for women with RA after 8 weeks had no significant differences in total antioxidant capacity, oxidized low density lipoprotein, malondialdehyde and high sensitivity C-reactive protein compared to placebo groups (Javadi et al., 2014). The clinical trials of quercetin for the treatment of RA were shown in Table 4.

**TABLE 4:** An overview on clinical trials evaluating effects of compounds involved in EH on RA.

NO.	Flavonoids	Dose	Duration	Size	Results	References
1	quercetin	500 mg/day	8 weeks	50	↓hs-TNFα, EMS, pain, DAS-28, HAQ	Javadi et al. (2017)
2	quercetin	500 mg/day	8 weeks	51	no significant differences in TAC, ox-LDL, MDA, hs-CRP	Javadi et al. (2014)
3	quercetin (plus azathioprine)	1,500 mg/day	8 weeks	190	↓ICAM-1, IL-6, complement proteins, ↑IL-10	Al-Rekabi et al. (2015)

hs-TNFα, high sensitivity tumor necrosis factor α, EMS, early morning stiffness; DAS-28, Disease Activity Score–28; HAQ, health assessment questionnaire; ICAM-1, intercellular adhesion molecule I; TAC, total antioxidant capacity; ox-LDL, oxidized low density lipoprotein; MDA, malondialdehyde; ↑ = up-regulation; ↓ = down-regulation.

## Alternative strategies for the treatment of RA

### Combination with approved drugs

According to European League Against Rheumatism (EULAR) recommendation, DMARDs should be started as soon as the diagnosis of RA (Smolen et al., 2020). Methotrexate (MTX), an efficacious DMARDs, is the first line for the treatment of RA. However, some adverse effects of MTX have limited their extensive clinical application, such as liver dysfunction, renal failure and nausea (Katturajan et al., 2021). In order to overcome the disadvantages and further improve the effective in treatment, it is necessary to explore combination therapy of compounds from EH and DMARDs for the treatment of RA.

Compared to the quercetin (30 mg/kg orally) or MTX (0.75 mg intraperitoneally twice a week) groups, the combination therapy significantly inhibited paw thickness and expression of proinflammatory cytokines including IL-1β, TNF-α, IL-6, and IL-17 in CIA mice (Haleagrahara et al., 2018). Moreover, quercetin reversed the transaminases levels of MTX groups including alanine aminotransferase (ALT) and aspartate aminotransferase (AST) (Costa et al., 2021). However, the other report showed that the concurrent therapy of quercetin (30 mg/kg orally) and MTX (0.5 mg/kg intraperitoneally) did not provide greater protection than a single agent (Haleagrahara et al., 2017).

### Bioavailability improvement

**FIGURE 4:**
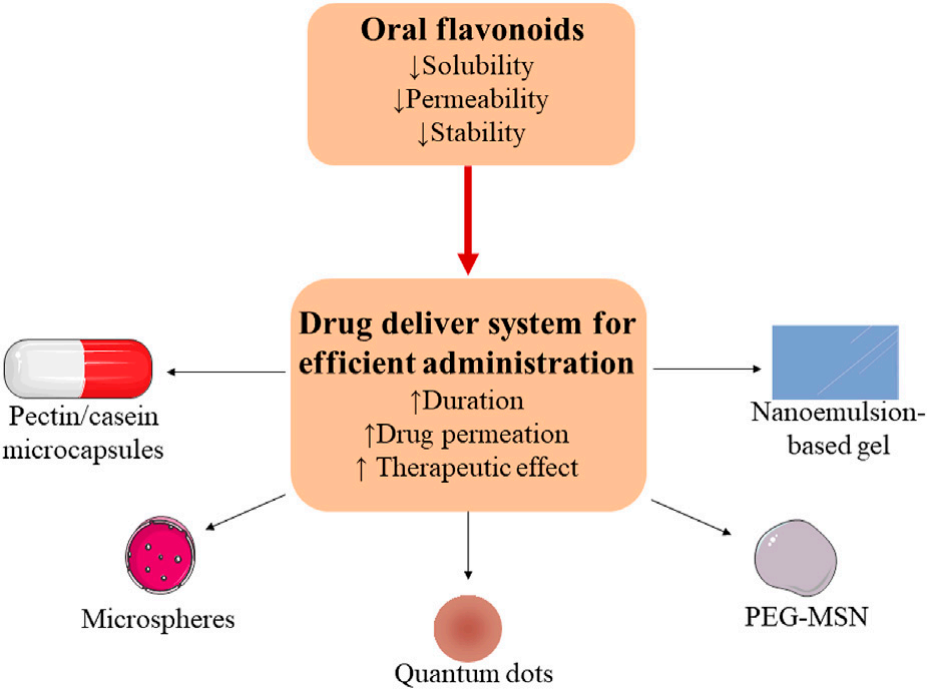
Limitations of flavonoids through oral administration in RA therapy and their delivery systems for a promising option.

EH has been commonly used in various traditional Chinese decoctions for the treatment of RA. Compared to other delivery routes, oral administration has clear advantages including lower pain and less risk of cross-infection (Das and Chaudhury, 2011). Consequently, oral administration was frequently chosen as the primary clinical administration approach. However, poor solubility, low permeability and inferior stability by oral administration result in restriction of their effectiveness (Zhao et al., 2019). For example, bioavailability of quercetin is pharmaceutically characterized as poor solubility, low bioavailability, poor permeability and instability (Cai et al., 2013). Furthermore, low concentration of those components by oral administration exerts curative effects in the joint cavities with systemic circulation. Given the above characteristics and disadvantages, new technologies have drawn increased attention to improve the bioavailability and effectiveness of the components. The new technologies of bioavailability improvement of EH were shown in Figure 4.

Nanoencapsulation technology including liposomes, nanoemulsions, and nanocapsules appear to be a promising option to increase bioavailability of the compounds (Han et al., 2020). Treatment of 10 mg/kg quercetin carried by pectin/casein microcapsules reduced oxidative stress and had no hepatotoxicity and mitochondriotoxicity by oral administration in AIA rats (Souza et al., 2021). *In vitro* and *in vivo* studies reported that quercetin loaded in polycaprolactone microspheres increased the duration in joint cavity for more than 30 days (Natarajan et al., 2011). Quercetin loaded by nanoemulsion-based gel had no toxic effect on synoviocytes and improved drug permeation and attenuated paw edema over 24 h in AIA rats (Gokhale et al., 2019). Complexes of quantum dots and quercetin significantly inhibited inflammation and oxidative stress and improved cartilage regeneration in AIA rats (Jeyadevi et al., 2013). Compared with luteolin treatment, combination of polyethylene glycol-mesoporous silica nano-carriers and luteolin was more effective and had longer existence time in AIA rats (Pang et al., 2021)*.*

## Safety assessment of EH

EH is an ancient traditional Chinese herbal for 2000 years and has been frequently used to treat various disease. However, it was reported that EH could cause drug-induced liver injury. Wang et al. found that water extract and alcohol extract of *Epimedium brevicornu* Maxim (20, 40, 80 g/kg) for 8 weeks reduced body weight of mice. Furthermore, the organ coefficient, blood indicators, serum biochemical indicators had a certain degree of change (*P*＜0.05), indicating that EH at 140 times the maximum human dose have certain toxic effects on mice for 8 weeks (Wang. et al., 2018). *In vivo* study showed that *Epimedium koreanum* Nakai had liver toxicity which was enhanced by increased dosage and prolonged time (Zhang. et al., 2018). Therefore, although EH is relatively safe for clinical use, it is still necessary to be cautious and do not take large doses or take it for a long time.

Previous research has reported that quercetin at dietary intake levels did not exert harmful effects on human health (Harwood et al., 2007). Recent study also showed that no zebrafish died or had abnormal morphology under 200 μM icariin (Zhong et al., 2019).

*In vivo* studies reported that hyperoside (65 and 500 mg/kg) had chronic hepatotoxicity and nephrotoxicity in beagle dogs, but the destruction was reversed and returned to normal after withdrawal (Ai. et al., 2015). High concentrations (36 μg/ml) of ikarisoside A induced liver injury in HL-7702 and HepG2 Cells *via* enhancing oxidative stress and inducing apoptosis (Zhang L. et al., 2019).

## Conclusion and future perspectives

RA is a chronic autoimmune disease and causes of RA are unclear. In TCM theory, the patients with RA are affected by “Wind”, “Cold”, “Damp” and “Kidney deficiency”. EH can strengthen bones and muscle, dispel wind chill and tonify the kidney, so it has been widely used in prescriptions of TCM for the treatment of RA. Remarkable curative effects in clinic draw increased attention and pharmacological research to investigate material foundation and pharmacological mechanism.

In this review, 293 components were searched from EH in different database and 15 compounds were filtered for the treatment of RA. Then, therapeutic effect, pharmacological mechanism, bioavailability and toxicity of the components were overall summarized and analyzed. This paper summarized the mechanism of the compounds in the treatment of RA through studies *in vivo* and *in vitro*. Studies showed that the components from EH have extensive pharmacological activities including anti-inflammatory, immunoregulatory, antioxidant, antiangiogenic, anti-FLS and osteoprotective effects.

**FIGURE 5:**
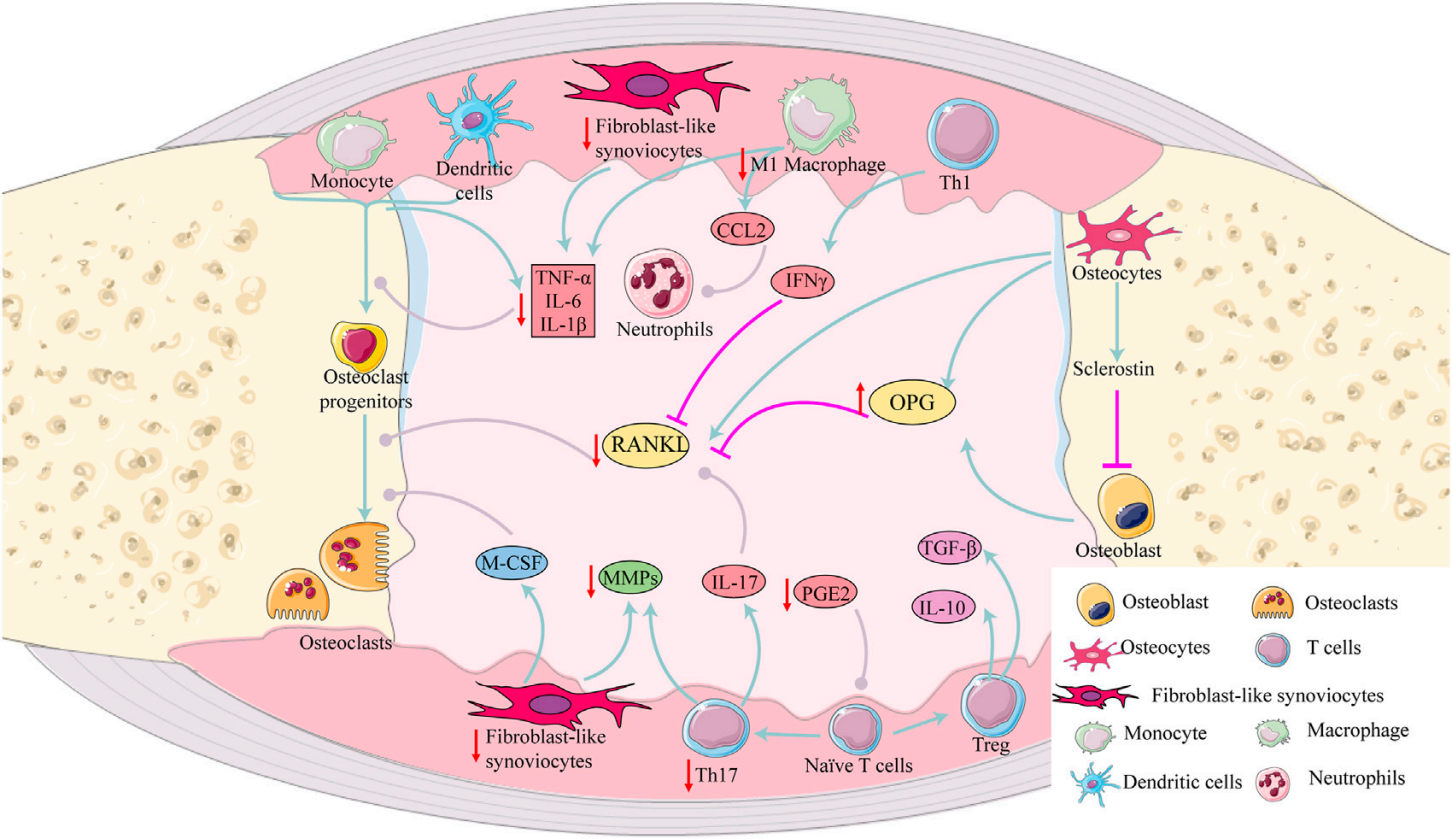
A schematic view of anti-RA effects of icariin (↑ = up-regulation; ↓ = down-regulation; red arrow = icariin) (images cited from Servier Medical Art images at www.servier.com).

By summarizing, it is no wonder that icariin is prime component and one of chemical markers for quality control of EH, which have bright particularly attention to the health-promoting effects. Icariin has anti-RA effects *via* anti-inflammatory, immunoregulatory, osteoprotective and antioxidant activities. A schematic view of anti-RA effects of icariin was shown in Figure 5. However, most of data were acquired from laboratory tests in several animal and cellular models. Clinical trials are required to carried out to explore the possible therapeutic effects of icariin for the management of RA.

A large number of components from EH were searched and the results indicate that EH may have the multicomponent and multifunctional traits in the treatment of RA. The mechanism of compounds from EH for the treatment of RA was shown in Table 5. However, there are few published studies exploring the anti-RA effects of other compounds isolated from EH. In this review, anti-RA effects of the compounds in other species were inferred to EH. Therefore, it is necessary to investigate the anti-RA effects of other compounds from EH in the future.

**TABLE 5:** The mechanism of compounds from EH for the treatment of RA.

Compounds	Anti-inflammatory activities	Immunoregulatory activities	Osteoprotective activities	FLS		Antiangiogenic activities	Antioxidant activities
Apigenin	↓TNF-α, IL-12p70, IL-10, CXCR4, COX2, NO, iNOS	↓Th1 cells, DCs maturation	↓RANKL, RANK, MMP3	↓proliferation, PI3K/Akt		↓VEGF, VEGFR1, VEGFR2, VCAM	↑ROS
	↑IL-1β	↑Th2 cells	↑OPG	↑apoptosis, miR-223-3p			
Astragalin	↓TNF-α, IL-1β, IL-6, IL-8		↓MMP-1, MMP-3, MMP-13, MAPKs, c-Jun/AP-1				
Chlorogenic acid		↓Th1 cells, BAFF, NF-κB		↓proliferation, NF-κB, JAK/STAT			
		↑Th2 cells		↑apoptosis			
Emodin	↓TNF-α, IL-6, PGE2	↓NETs	↓MMP-1, MMP-3, Osteoclastogenesis, NF-κB				
	COX-2	↑neutrophil apoptosis					
Hyperoside	↓TNF-α, IL-6, iNOS		↓MMP-3, MMP-9	↓proliferation, migration, NF-κB			
Icariin	↓IL-1β, IL-6 and TNF-α, PGE2	↓Th17 cells, M1 macrophage	↓RANKL, MMP-9	↓migration, proliferation			↓mitochondrial transmembrane potential
		↑M2 macrophage	↑OPG	↑apoptosis			↑ROS
Ikarisoside A			↓MMP9, NF-κB, JNK, Akt, c-Fos, NFATc1				
Isoliquiritigenin	↓IL-1β, iNOS, COX2		↓osteoclastogenesis, MAPK, NF-κB				
Kaempferitrin	↓IL-1β, IL-6, TNF-α		↓MMP1, MMP3	↓proliferation, NF-κB, Akt/mTOR			
				↑apoptosis			
Kaempferol	↓IL-17, IL-21, TNF-α, IL-6, IL-1β, NO, iNOS, COX-2, PGE2	↓Th17 cells	↓MMP1, MMP3	↓migration, proliferation, MAPK, NF-κB			↓ROS myeloperoxidase
		↑Treg cells		↑apoptosis			
Luteolin	↓TNF-α, IL-1β, NO, iNOS, COX-2		↓MMP1, MMP3, RANKL, MAPKs, AP-1, NF-κB, PI3K-Akt	↓proliferation, NF-κB, JAK/STAT, PI3K-Akt	↓VEGF, HIF-1α		↓Malondialdehyde, 15-lipoxygenase
			↑OPG	↑apoptosis			
Magnoflorine	↓IL-6, IL-8, iNOS			↓migration, invasion, proliferation, Keap1, PI3K/Akt/NF-κB			
	↑TNF-α, IL-1β			↑apoptosis, cell cycle arrest, Nrf2, HO-1			
Quercetin	↓IL-1β, IL-6, TNF-α, IL-8, IL-13, IL-17, MCP-1, PGE2, COX2, NO, iNOS	↓Th17 cells, neutrophil activity	↓MMP1, MMP3, MMP-13 RANKL	↓migration, NF-κB	↓VEGFA, HIF-1α		↓ROS, TBARS, 12/15-lipoxygenase, myeloperoxidase
		↑Treg cells		↑apoptosis			
Tricin	↓TNF-α, IL-6, IL-1β						
β-Sitosterol		↓M1 macrophage			↓VEGFR2, p-VEGFR2		
		↑M2 macrophage					
